# Investigating the role of common and rare variants in multiplex multiple sclerosis families reveals an increased burden of common risk variation

**DOI:** 10.1038/s41598-022-21484-x

**Published:** 2022-10-10

**Authors:** Elif Everest, Mohammad Ahangari, Ugur Uygunoglu, Melih Tutuncu, Alper Bulbul, Sabahattin Saip, Taskin Duman, Ugur Sezerman, Daniel S. Reich, Brien P. Riley, Aksel Siva, Eda Tahir Turanli

**Affiliations:** 1 Department of Molecular Biology and Genetics, Faculty of Science and Letters, Istanbul Technical University, Maslak, Istanbul, Turkey; 2 Department of Psychiatry, Virginia Institute for Psychiatric and Behavioral Genetics, Virginia Commonwealth University, Richmond, VA USA; 3 Department of Neurology, Cerrahpasa School of Medicine, Istanbul University-Cerrahpasa, Fatih, Istanbul, Turkey; 4 Department of Biostatistics and Bioinformatics, Institute of Health Sciences, Acibadem University, Atasehir, Istanbul, Turkey; 5 Department of Neurology, Tayfur Ata Sokmen School of Medicine, Mustafa Kemal University, Alahan-Antakya, Hatay, Turkey; 6 Department of Biostatistics and Medical Informatics, Faculty of Medicine, Acibadem University, Atasehir, Istanbul, Turkey; 7 Translational Neuroradiology Section, National Institute of Neurological Disorders and Stroke, National Institutes of Health, Bethesda, MD USA; 8 Department of Psychiatry and Human and Molecular Genetics, Virginia Institute for Psychiatric and Behavioral Genetics, Virginia Commonwealth University, Richmond, VA USA; 9 Department of Molecular Biology and Genetics, Faculty of Engineering and Natural Sciences, Acibadem University, Atasehir, Istanbul, Turkey; 10 Molecular and Translational Biomedicine Program, Graduate School of Natural and Applied Sciences, Acibadem University, Atasehir, Istanbul, Turkey

**Keywords:** Multiple sclerosis, Medical genomics

## Abstract

Many multiple sclerosis (MS)-associated common risk variants as well as candidate low-frequency and rare variants have been identified; however, approximately half of MS heritability remains unexplained. We studied seven multiplex MS families, six of which with parental consanguinity, to identify genetic factors that increase MS risk. Candidate genomic regions were identified through linkage analysis and homozygosity mapping, and fully penetrant, rare, and low-frequency variants were detected by exome sequencing. Weighted sum score and polygenic risk score (PRS) analyses were conducted in MS families (24 affected, 17 unaffected), 23 sporadic MS cases, 63 individuals in 19 non-MS control families, and 1272 independent, ancestry-matched controls. We found that familial MS cases had a significantly higher common risk variation burden compared with population controls and control families. Sporadic MS cases tended to have a higher PRS compared with familial MS cases, suggesting the presence of a higher rare risk variation burden in the families. In line with this, score distributions among affected and unaffected family members within individual families showed that known susceptibility alleles can explain disease development in some high-risk multiplex families, while in others, additional genetic contributors increase MS risk.

## Introduction

Multiple sclerosis (MS) is a chronic, neuroinflammatory, neurodegenerative disease of the central nervous system with both genetic and environmental risk factors. Twin and family studies support a genetic component for MS^1–3^, and early genetic analyses revealed the association of MS with the major histocompatibility complex (MHC) region^4,5^. Subsequently, hundreds of MS-associated common risk variants with low-to-moderate effect sizes in MHC and non-MHC regions have been identified mainly through genome-wide association studies (GWAS)^6,7^. A meta-analysis conducted by the International MS Genetics Consortium (IMSGC) involving 47,351 MS cases and 68,284 healthy controls identified 32 MHC, 200 non-MHC, and 1 X-linked loci associated with MS risk^8,9^. However, these 233 loci together can explain only about 50% of expected MS heritability^9^.

In addition to common variants, a number of low-frequency and rare variants have been associated with MS risk through candidate gene analyses^10^ and exome sequencing^11^. Mitrovič et al. conducted a meta-analysis on 32,367 MS cases and 36,012 controls to identify MS-associated low-frequency and rare variants and found that as much as 5% of the heritability can be explained by low-frequency variants in coding regions^12^, still leaving a large proportion of MS heritability unexplained. In this study, we studied seven multiplex MS families from eastern Turkey, where consanguineous marriage rates are as high as 42.6%^13^. We searched for rare and low-frequency, high-penetrant variants segregating within the families and conducted weighted sum score and polygenic risk score (PRS) analyses to elucidate the role of common risk variation in the increased risk of MS in these seven families.

## Results

### Linkage analysis, homozygosity mapping, and exome sequencing

**Figure 1:**
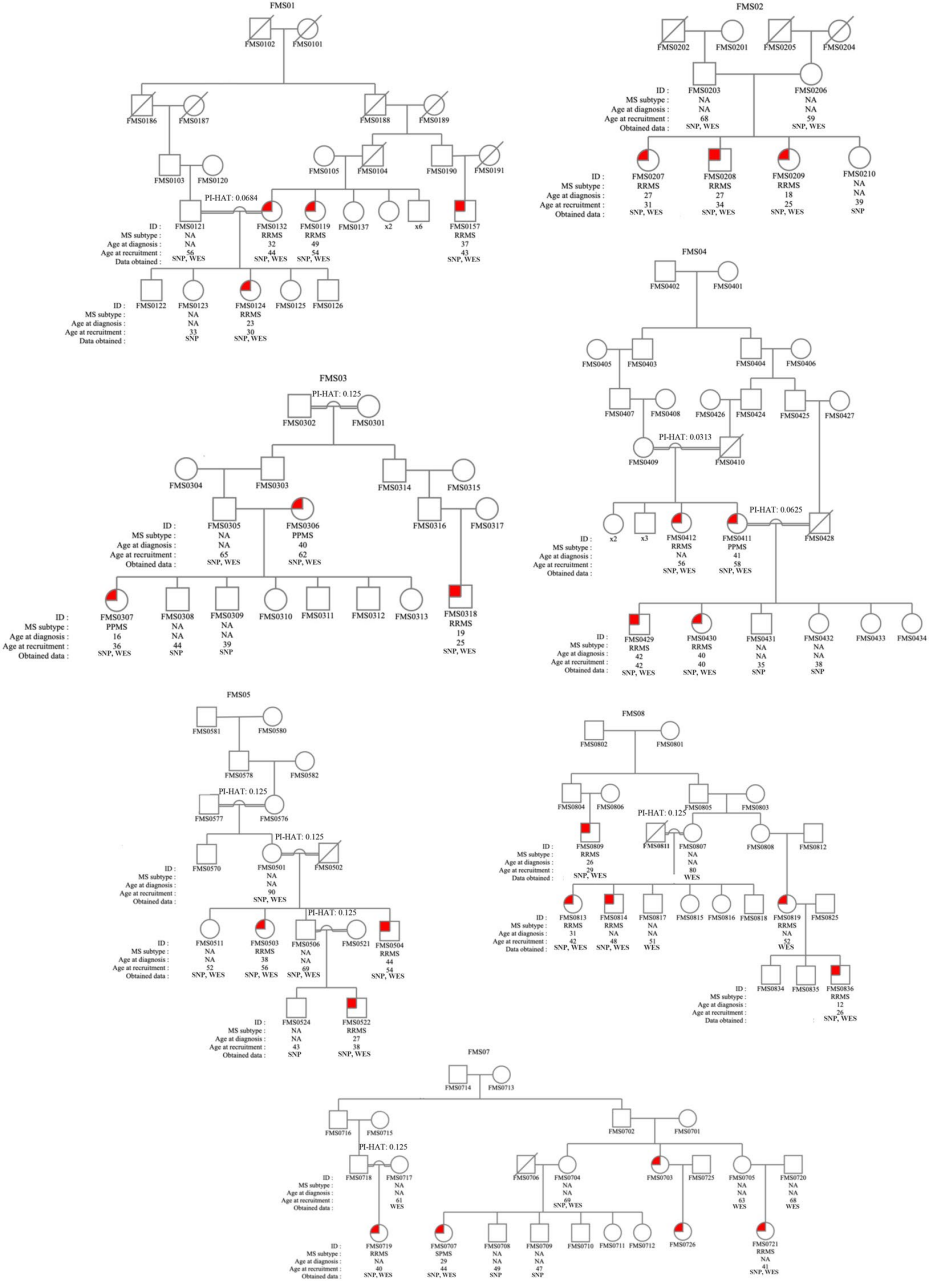
Simplified pedigrees of MS families included in the study. Males are represented by squares and females by circles. Diagonal line, deceased individual; red-colored symbol, clinically and magnetic resonance imaging-proven MS case; open symbol, non-MS relative. Individuals for whom SNP genotyping or exome sequencing was done are indicated with “SNP” and “WES,” respectively. Double line indicates consanguineous marriage, and relatedness degrees between these individuals are shown as PI-HAT values. PI-HAT for FMS0121 and FMS0132 was calculated using PLINK. Values for other consanguineous couples indicate the expected PI-HAT values based on the reported family relationships by the study participants. Pedigrees were constructed using the genetic data management system, Progeny Clinical—Web Version 9 from Progeny Genetics (Copyright 2019. Reprinted with permission of Progeny Genetics LLC, Delray Beach, FL, www.progenygenetics.com). NA, not applicable; PPMS, primary progressive MS; RRMS, relapsing–remitting MS; SPMS, secondary progressive MS.

**Figure 2:**
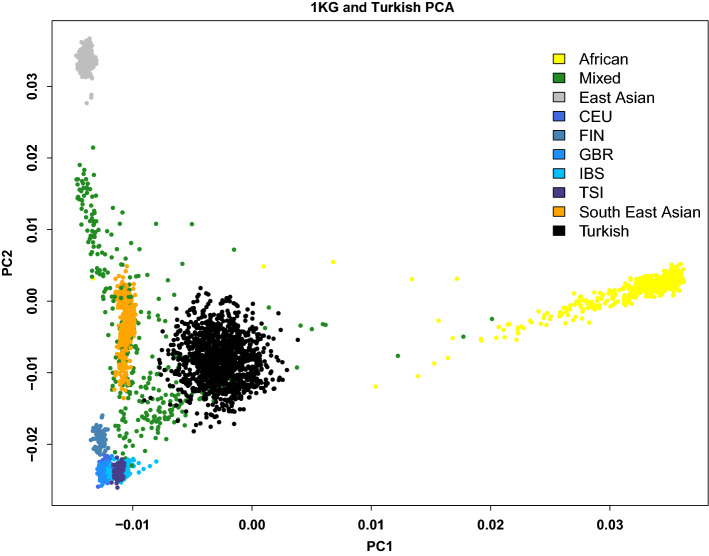
Continental principal component analysis (PCA) of the Turkish samples (black color) projected on the 1000 Genomes Phase 3 data shows that Turkish samples cluster closely together as a mixed population. PC1 on the X-axis and PC2 on the Y-axis. Each color represents one of the ancestral groups. Abbreviations: CEU, Northern Europeans in Utah; FIN, Finnish in Finland; GBR, British in England and Scotland; IBS, Iberian populations in Spain; TSI, Tuscans in Italy.

Pedigrees of the seven families studied are shown in Fig. 1. All cases were clinically examined and had their MS diagnosis confirmed with MRI according to the McDonald 2017 criteria^14^. Among those, 21 cases had relapsing–remitting MS, and 4 had progressive MS. The mean age was 42 ± 10.94 years in the MS group and 55.36 ± 15.23 in the unaffected family members (*P* = 0.0011). The female-to-male ratio was 1.78 in the MS cases. In the sporadic MS group, 20 cases had relapsing–remitting MS, and 3 had progressive MS. The mean age was 41.39 ± 8.36, and the female-to-male ratio was 1.56 in this group. There were no significant differences in demographic characteristics between familial and sporadic MS cases. Principal component analysis (PCA) of the samples showed that all participants were of Turkish origin and closely clustered together as a mixed population (Fig. 2).

SNP genotypes (710 K or 2.5 M, Illumina) were obtained for all familial cases whose DNA samples were available (N = 24) and for the oldest healthy family members possible (N = 17). Non-parametric linkage analysis yielded 183 regions with LOD scores higher than 1.2 in four of the families (Supplementary Table S1 and Supplementary Figure S1). Homozygosity mapping resulted in a total of 114 homozygous regions of > 200 kb in size that were exclusively shared by the MS cases within families (Supplementary Table S2). Exome sequencing analysis was performed, including MS cases (N = 25) and unaffected family members older than 50 years of age (N = 13), revealing 42 heterozygous and 1 homozygous fully penetrant, rare or low-frequency, exonic variants (Supplementary Table S3). All variants were rare or low-frequency both in the gnomAD (all populations) and Turkish population based on the work by Kars et al^15^. Thirty of the heterozygous variants were located in the candidate linkage regions in family FMS01, and the homozygous variant detected in family FMS02 was located in the homozygous region with a size of 539 kb and a LOD score of 1.2 (Table 1). Other variants were segregated in families FMS03, FMS04, and FMS05 (Supplementary Table S3), while there were no such variants in families FMS07 and FMS08.

**Table 1:** Fully penetrant, rare and low-frequency, exonic variants located in the candidate linkage and homozygous regions.

Family	Chr	Location (GRCh37)	Ref	Alt	Gene	Status	Frequency in gnomAD (all-exome)	Frequency in the Turkish population*	LOD Score
FMS01	1	43905286	C	T	*SZT2*	Heterozygous	0.0200	0.0152	1.204
FMS01	1	54534492	A	G	*TCEANC2*	Heterozygous	0.0002	0.0023	1.203
FMS01	1	54645000	G	A	*CYB5RL*	Heterozygous	0.0000	NA	1.203
FMS01	2	11758697	C	T	*GREB1*	Heterozygous	0.0001	NA	1.204
FMS01	4	36093506	C	T	*ARAP2*	Heterozygous	0.0006	0.0016	1.204
FMS01	5	31532534	G	C	*C5orf22*	Heterozygous	0.0056	0.0057	1.204
FMS01	5	121356311	G	A	*SRFBP1*	Heterozygous	0.0001	0.0011	1.204
FMS01	5	140307358	A	G	*PCDHAC1*	Heterozygous	0.0002	0.0018	1.204
FMS01	5	140554310	G	T	*PCDHB7*	Heterozygous	0.0063	0.0039	1.204
FMS01	5	140563158	G	C	*PCDHB16*	Heterozygous	0.0066	0.0071	1.204
FMS01	5	140567496	C	G	*PCDHB9*	Heterozygous	0.0010	0.0012	1.204
FMS01	5	140572841	C	T	*PCDHB10*	Heterozygous	0.0066	0.0068	1.204
FMS01	5	140590181	G	A	*PCDHB12*	Heterozygous	0.0061	0.0032	1.204
FMS01	5	141242727	C	T	*PCDH1*	Heterozygous	0.0004	0.0002	1.204
FMS01	7	102113188	C	T	*LRWD1*	Heterozygous	0.0020	0.0094	1.204
FMS01	7	105665004	C	A	*CDHR3*	Heterozygous	0.0085	0.0091	1.204
FMS01	8	120612927	G	A	*ENPP2*	Heterozygous	0.0001	NA	1.204
FMS01	8	121220518	G	A	*COL14A1*	Heterozygous	0.0024	0.0015	1.203
FMS01	8	133584564	G	A	*LRRC6*	Heterozygous	0.0014	0.0037	1.204
FMS01	10	27462061	G	A	*MASTL*	Heterozygous	0.0159	0.0240	1.204
FMS01	14	21896304	C	T	*CHD8*	Heterozygous	0.0002	0.0019	1.204
FMS01	18	47363963	T	C	*MYO5B*	Heterozygous	0.0234	0.0168	1.204
FMS01	18	61255916	C	T	*SERPINB13*	Heterozygous	0.0000	0.0004	1.202
FMS01	18	61584726	G	T	*SERPINB10*	Heterozygous	0.0055	0.0114	1.201
FMS01	19	4311946	C	T	*FSD1*	Heterozygous	0.0009	NA	1.203
FMS01	19	6456458	G	A	*SLC25A23*	Heterozygous	0.0006	0.0012	1.203
FMS01	19	6475303	G	A	*DENND1C*	Heterozygous	0.0000	0.0006	1.203
FMS01	19	10273374	T	G	*DNMT1*	Heterozygous	0.0001	0.0007	1.203
FMS01	19	15288695	G	A	*NOTCH3*	Heterozygous	0.0000	NA	1.203
FMS01	19	16918662	G	A	*NWD1*	Heterozygous	0.0070	0.0061	1.203
FMS02	7	100677893	C	A	*MUC17*	Homozygous	0.0206	0.0397	1.204

*NA* not available.

*Variant frequencies in the Turkish population based on the work by Kars et al.^15^.

### Sum score and polygenic risk score analyses

**Figure 3:**
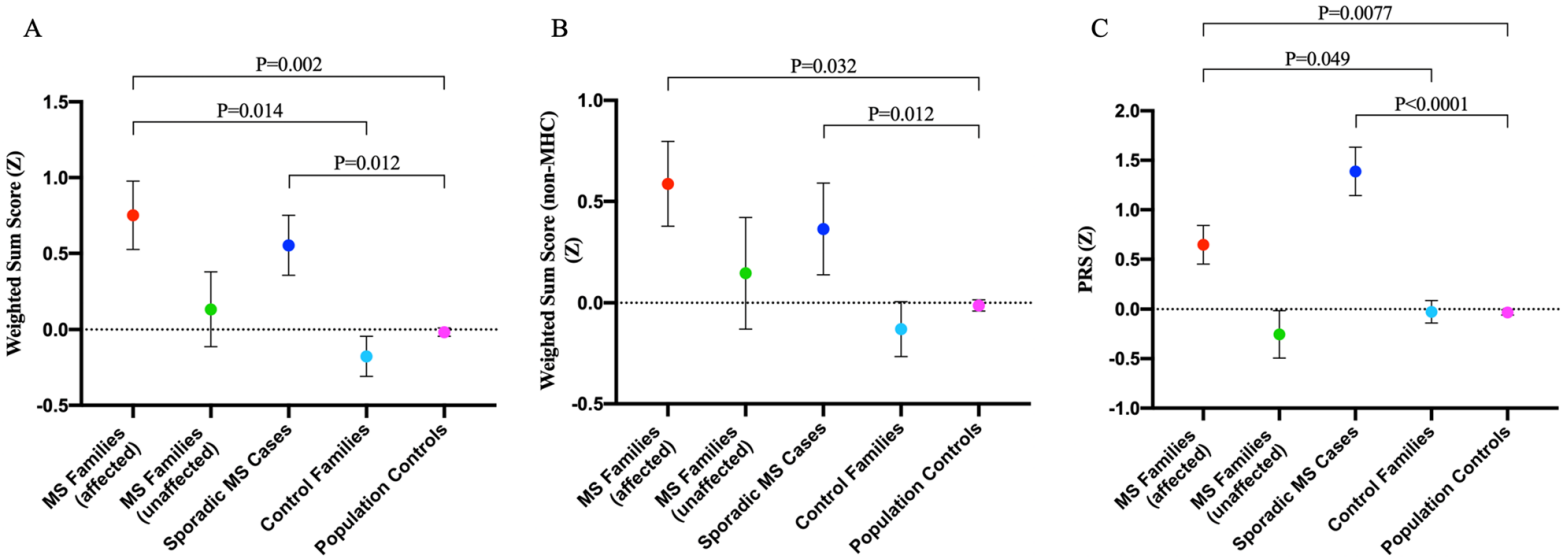
Weighted sum scores and polygenic risk scores (PRS) are higher in the affected members of MS families compared with the control families and healthy population controls. **(A)** Comparisons of weighted sum scores that include both MHC and non-MHC alleles among the study groups. **(B)** Comparisons of weighted sum scores that include only non-MHC alleles among the study groups. **(C)** Comparisons of PRS values among the study groups. Logistic regression analysis. Data are mean ± s.e.m. *P* < 0.05, significant.

Logistic regression analyses showed that the weighted sum scores of MS cases were significantly higher than those of the population controls and control families (Fig. 3A, *P* = 0.002 and *P* = 0.014, respectively, after Bonferroni correction). When we excluded the MHC alleles from the sum score calculation, the difference between MS cases and healthy population controls remained significant (Fig. 3B, *P* = 0.032, after Bonferroni correction); however, the decreased significance level indicates that the MHC region adds more burden on the affected family members in these families. There was also a significantly higher burden of MS PRS in the affected members of MS families compared with both healthy population controls and control families (Fig. 3C, *P* = 0.0077 and *P* = 0.049, respectively, after Bonferroni correction). Higher PRS in the affected individuals increased the MS risk by OR = 1.84 and OR = 2.27 in MS cases compared with the population controls and control families, respectively (Table 2). Higher weighted sum scores that included the MHC alleles increased the risk by OR = 2.16 and OR = 2.4 in MS cases compared with the population controls and control families, respectively, which decreased to 1.83 and 1.92 when the MHC alleles were excluded (Table 2). Sporadic MS cases had higher but non-significant PRS compared with familial MS cases (*P* = 0.087) and control families (*P* = 0.058) after Bonferroni correction, while this observation was significant compared with population controls (*P* = 5.31E−09) (Fig. 3C). There was no difference in sum scores of familial and sporadic MS cases (Fig. 3A, *P* = 0.95; Fig. 3B, *P* = 0.93).

**Table 2:** Association of polygenic risk scores and weighted sum scores with risk of MS.

Genetic risk score	Comparison	OR	Lower CI	Higher CI	Adjusted *P* value
Polygenic risk score	Familial MS cases versus Population controls	1.84	1.277	2.63	0.0077*
	Familial MS cases versus Control families	2.27	1.323	4.318	0.0491*
	Familial MS cases versus Unaffected relatives	2.61	1.322	6.085	0.1053
	Sporadic MS cases versus Familial MS cases	1.83	1.11	3.7	0.087
Weighted sum score	Familial MS cases versus Population controls	2.16	1.439	3.279	0.002*
	Familial MS cases versus Control families	2.4	1.447	4.327	0.014*
	Familial MS cases versus Unaffected relatives	1.64	0.909	3.16	1
	Sporadic MS cases versus Familial MS cases	0.82	0.45	1.44	0.95
Weighted sum score (non-MHC)	Familial MS cases versus Population controls	1.83	1.222	2.771	0.0324*
	Familial MS cases versus Control families	1.92	1.193	3.276	0.0972
	Familial MS cases versus Unaffected relatives	1.4	0.793	2.613	1
	Sporadic MS cases versus Familial MS cases	0.81	0.45	1.4	0.93

OR, odds ratio (95% confidence intervals [CI]); Adjusted *P* value, after Bonferroni correction; *, significant.

**Figure 4:**
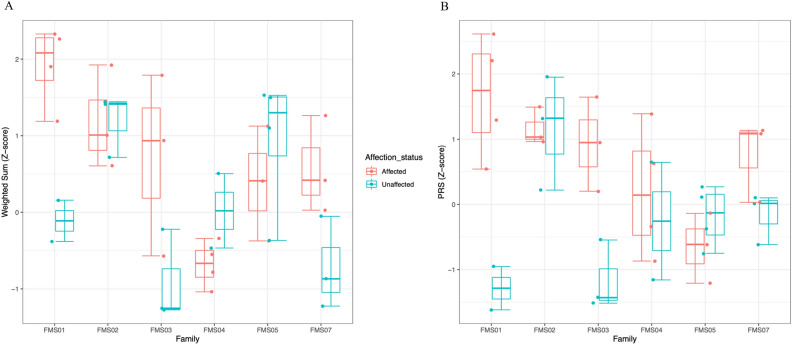
Box plots showing the weighted sum score and polygenic risk score (PRS) distributions in MS families. **(A)** Weighted sum scores including both MHC and non-MHC alleles of each affected and unaffected member in MS families. **(B)** PRS of each affected and unaffected member in MS families. Each data point represents the genetic risk score of an individual. Boxes are mean ± 1.5 × interquartile range.

Although the affected individuals in the MS families had apparently higher weighted sum score and PRS values compared with the unaffected family members, the differences were not significant after Bonferroni correction (Table 2). When looking at individual families, the higher sum score and PRS trend in MS cases was only observed in three of the families (Fig. 4, families FMS01, FMS03, and FMS07). This pattern was not observed in families FMS02, FMS04, and FMS05, in which there were no apparent differences in the sum score and PRS values between the affected and unaffected family members or the unaffected individuals had higher sum score and PRS values compared with their relatives with MS (Fig. 4). The intra-family comparison could not be done for family FMS08 since no DNA samples from the healthy family members were available for SNP genotyping. Weighted sum score values calculated with both MHC and non-MHC alleles and PRS values for each individual are shown in Table 3.

**Table 3:** Weighted sum score and polygenic risk score of each family member.

Family ID	Individual ID	Disease status	Weighted sum score*	Polygenic risk score	Age^#^
FMS01	FMS0119	Affected	1.57	1.35	54
	FMS0124	Affected	2.19	2.28	30
	FMS0132	Affected	2.26	2.70	44
	FMS0157	Affected	1.25	0.58	43
	FMS0121	Unaffected	0.09	− 1.61	56
	FMS0123	Unaffected	− 0.45	− 0.94	33
FMS02	FMS0207	Affected	1.21	1.02	31
	FMS0208	Affected	2.12	1.55	34
	FMS0209	Affected	0.80	1.09	25
	FMS0203	Unaffected	1.64	2.02	68
	FMS0206	Unaffected	0.78	0.26	59
	FMS0210	Unaffected	1.48	1.38	39
FMS03	FMS0306	Affected	− 0.63	1.00	62
	FMS0307	Affected	0.87	0.24	36
	FMS0318	Affected	1.59	1.71	25
	FMS0305	Unaffected	− 0.16	− 1.42	65
	FMS0308	Unaffected	− 1.18	− 1.51	44
	FMS0309	Unaffected	− 1.21	− 0.52	39
FMS04	FMS0411	Affected	− 0.28	0.67	58
	FMS0412	Affected	− 0.72	1.45	56
	FMS0429	Affected	− 1.10	− 0.85	42
	FMS0430	Affected	− 0.61	− 0.32	40
	FMS0431	Unaffected	0.57	0.69	35
	FMS0432	Unaffected	− 0.40	− 1.15	38
FMS05	FMS0503	Affected	0.61	− 1.20	56
	FMS0504	Affected	1.32	− 0.59	54
	FMS0522	Affected	− 0.17	− 0.11	38
	FMS0501	Unaffected	1.30	0.31	90
	FMS0506	Unaffected	1.72	0.15	69
	FMS0511	Unaffected	1.69	− 0.35	52
	FMS0524	Unaffected	− 0.17	− 0.73	43
FMS07	FMS0707	Affected	0.62	1.19	44
	FMS0719	Affected	− 0.04	1.14	40
	FMS0721	Affected	1.20	0.07	41
	FMS0704	Unaffected	0.01	− 0.59	69
	FMS0708	Unaffected	− 0.93	0.13	49
	FMS0709	Unaffected	− 1.16	0.05	47

*Weighted sum scores calculated including both MHC and non-MHC alleles.

^#^Age information at the time of inclusion in the study.

### Correlation among the genetic, clinical, and radiological data

All cases included in this study had their MS diagnosis confirmed with MRI. Even though all four MS cases in family FMS01 had higher sum score and PRS values compared with their two healthy relatives (Fig. 4 and Table 3), we have detected 26 fully penetrant, rare/low-frequency heterozygous variants, 18 of which are located in the candidate linkage regions (Table 1 and Supplementary Table S3). Among those, there were variants located in genes that involve in the immune system (e.g., *CDHR3*) and nervous system (e.g., *TCEANC2*), suggesting a possible role of one or a combination of these variants in increased MS risk together with the accumulation of common MS-associated variants in this family.

In family FMS02, two healthy family members had a higher common risk variation burden than their affected relatives (Fig. 4 and Table 3, individuals FMS0203 and FMS0210). FMS0203, the father, is a 68-year-old man whose MRI examination revealed no significant findings. MRI examination for the 39-year-old sister (FMS0210) of the affected individuals could not be concluded due to claustrophobia. Neither has any MS-suggestive health issues. Clinical and radiological findings of this family suggest that there may be other risk variants increasing the MS risk in the affected members of the family. We have detected one fully penetrant, homozygous, low-frequency variant (rs78263695, p.P1066T) in the *MUC17* gene (Table 1), which encodes for mucin-17 and is associated with inflammatory conditions of the colon^16,17^. Rare variants in *MUC17* are also weakly associated with myelitis (SKAT *P* = 0.0132) and optic neuritis (SKAT *P* = 0.0384) in Genebass (https://genebass.org/), suggesting that *MUC17* rs78263695 variant, possibly together with other incomplete-penetrant variants, may modify MS risk in this family.

In families FMS03 and FMS07, MS cases had higher scores than their unaffected relatives (Fig. 4 and Table 3), suggesting that the increased MS risk may be largely attributable to the common MS-associated variants in the two families. Nevertheless, 12 rare/low-frequency variants outside the candidate regions were detected in FMS03 (Supplementary Table S3), some of which may contribute to MS risk in this family. In FMS04, one healthy family member (FMS0431), a 35-year-old man, had a higher common risk variation burden than his affected family members (Table 3). His neurological examination could not be performed; however, he later reported that he had experienced bilateral lower extremity numbness and weakness for three to four months approximately four years ago. He was not admitted to a neurology clinic for his symptoms, which faded without medication. Upon this, we requested an MRI scan of the brain and spinal cord, which did not disclose any significant findings, with the caveat that the spinal cord images were not of high quality. The individual will be followed to monitor possible changes in disease status. All four clinically and radiologically confirmed MS cases in this family share one low-frequency, heterozygous variant outside the candidate regions in the *PCNT* gene (rs12481791, p.A2433V), which encodes for pericentrin protein, an integral component of the pericentriolar material involving in microtubule organization during the cell cycle^18^. Mutations in the *PCNT* gene are responsible for autosomal recessive type 2 microcephalic osteodysplastic primordial dwarfism (OMIM #210720). *PCNT* is associated with a variety of other neurological symptoms both in humans and mice (http://www.informatics.jax.org/). The detected *PCNT* rs12481791 variant in family FMS04 has a frequency of 2.2% in the Turkish population and a CADD score of 23.8 and may influence MS risk through the nervous system involvement of the altered protein.

Sum score and PRS distributions in family FMS05 suggest that MS development cannot be largely attributable to the accumulation of common variants in the affected family members, although the only individual who may still be at MS risk in terms of age (FMS0524, 43-year-old) did not have a notably high sum score or PRS value (Fig. 4 and Table 3). Exome sequencing analysis revealed three rare, heterozygous variants with full penetrance in the *RNF217* (rs73580047), *PHLPP2* (rs148584091), and *TXNL4B* (rs780160954) genes in this family. *PHLPP2* codes a phosphatase that involves in Akt signaling and is associated with various cancers^19–21^. Thioredoxin Like 4B encoded by *TXNL4B* is involved in pre-mRNA splicing with no known strong disease associations. Rare variants in *RNF217* are associated with polyneuropathy, and rs73580047 (p.R457H) detected in FMS05 has a *P* value of 0.08 and a beta of 0.6 for this condition in Genebass. In addition, rare loss of function variants in the *RNF215* gene are weakly associated with MS (SKAT *P* = 0.017), suggesting a role of ring finger protein-coding gene variants in modifying the MS risk. In family FMS05, harboring these rare variants may have a low or moderate effect on increased MS risk. Within-family comparison of the sum score and PRS values could not be done for family FMS08 due to the absence of SNP data of healthy family members. Exome sequencing analysis revealed no candidate fully penetrant variants in families FMS07 and FMS08.

## Discussion

The contribution of a number of rare risk variants to the heritability of MS has been previously identified, largely by exome sequencing analyses in sporadic and familial MS cases^10,12,22^. However, the contribution of these rare variants, in combination with previously identified MS-associated common variants, can only explain about half of the MS heritability with current sample sizes. In this study, we collected multiplex MS families from the eastern parts of Turkey, where consanguineous marriage rates reach as high as 42.6%^13^, to reveal MS-associated genomic regions by linkage analysis and homozygosity mapping, high-risk rare/low-frequency variants by exome sequencing, and the effect of known MS-associated common variants in MS risk by genetic risk score analyses.

PRS analyses have been conducted and reported for schizophrenia^23,24^, coronary artery disease^25^, Alzheimer’s disease^26^, and cancer^27^. These analyses have successfully identified individuals at high risk for these polygenic conditions, which shows the potential predictive utility of PRS calculation in the future. In a recent study by Shams et al.^28^, higher PRS was shown to be associated with a significantly increased risk of developing MS from age 20 onwards and thalamic atrophy within 10 years of disease progression. To our knowledge, there is no study investigating the effect of genome-wide PRS using large genomic datasets on the risk of MS in family samples. The sum score approach, on the other hand, has been previously used to calculate the MS genetic burden, using selected sets of SNPs that are significantly associated with the risk of developing MS. In the Genes and Environment in Multiple Sclerosis (GEMS) project, environmental risk scores, weighted sum scores, and integrated genetic and environmental risk scores (GERS) were calculated in 1696 individuals with at least one first-degree relative with MS^29^. Sum score calculation included 64 MS-associated SNPs from the IMSGC study (2011)^7^ and revealed that asymptomatic subjects had significantly higher and lower GRS compared with healthy controls and MS cases, respectively. Later, 65 asymptomatic women (40 higher-risk and 25 lower-risk based on the GERS) from the GEMS project underwent neurological examination^30^. It was shown that women at higher risk had poorer vibration perception in the distal lower extremities. Moreover, four higher-risk women and one lower-risk woman had T2-weighted hyperintense brain lesions consistent with the 2010 McDonald MRI criteria^31^ for dissemination in space as well as other MRI features associated with MS, supporting the presence of a higher risk of developing MS in individuals with higher genetic burden and environmental risks. In a recent study, a weighted sum score calculation using 127 common risk variants from the IMSGC study (2011) and GWAS Catalogue was performed in singleton MS cases and controls from Orkney and Shetland populations^32^. It was shown that MS cases had significantly higher sum scores compared with the controls in each population, although there were no apparent differences among the three control populations, suggesting that the high MS prevalence in the Northern Isles of Scotland cannot be attributed to these common variants.

In this study, to understand the contribution of known susceptibility variants to the increased MS risk in our families, we determined the missing genotypes of the study participants whose SNP data were available through imputation and calculated the weighted sum score and genome-wide PRS for each individual (Supplementary Table S4). Overall, both weighted sum score and PRS values, as well as unweighted sum scores (Supplementary Table S5 and Supplementary Figure S2), were significantly higher in the affected members of the MS families compared with both healthy population controls and control families. Although not significant after the Bonferroni correction, PRS values of sporadic MS cases were higher than those of familial cases, suggesting the presence of higher rare risk variation loading in the families. In contrast, no significant difference in weighted sum scores was observed between familial and sporadic cases, possibly due to the high degree of convergence between common and rare risk variation in significant loci for MS. When sum scores and PRS were investigated within individual families, the values were higher in the affected members compared with their healthy relatives only in three of the families, further suggesting the polygenic inheritance of MS. In families that did not show this pattern, the detected fully penetrant, rare and low-frequency variants, possibly in combination with other incomplete-penetrant variants with low-to-moderate risk effects and environmental factors, may influence the risk of MS. Another possibility is that the currently healthy family members with high sum scores and PRS values may develop MS in the future or may have subclinical MS (“radiologically isolated syndrome”). However, most of the healthy family members with high scores were over 40 years old at the time their affection status was last confirmed and thus less likely to have new-onset clinical MS. One unaffected individual at the age of 35 with high sum score and PRS values later reported that he had experienced bilateral lower extremity numbness and weakness for three to four months four years prior, which fully recovered without medication, but his recent MRI scans did not disclose any significant findings.

Our data indicate that the increased burden of known disease-associated common MS risk variants and genome-wide PRS may explain disease development in some families, while the detected rarer variants may further modify MS risk in these families and others. The presence of complete and incomplete-penetrant, rare and low-frequency variants detected in these families, especially with the observed intra-familial discrepancies, should be further analyzed in other families to reveal whether they reach statistical significance for MS association. Future studies can reveal whether measurement of common risk variation burden is necessary for individuals at risk and whether these individuals should be followed with routine MRI scans.

There are several limitations to this study. First, even though the number of SNPs included in the weighted sum score analysis was higher than in previous sum score calculations in the literature, we were not able to include all 233 MS-associated SNPs identified by the IMSGC since some SNPs were imputed with low accuracy. This is in part because we could not impute all the MHC alleles efficiently due to the lack of a good reference panel for Turkish samples to impute the MHC region. Second, we have studied only seven families to investigate the ones with the highest number of affected family members and parental consanguinities. Another reason that we have selected those seven families is that we aimed to minimize the effect of environmental risk factor differences among the family members within individual families since members of each family have lived in the same area as their relatives. Due to the small sample size, we were unable to run mixed-model logistic regressions with genomic relationship to account for the relatedness of individuals in the families since the model did not converge. Finally, to confirm our observations and apply our hypotheses in a real-life setting, these findings should be replicated in studies with larger sample sizes.

## Methods

### Participants

A total of 25 MS cases and 22 unaffected family members in 7 families were included in the study. Six of the families had a family history of consanguineous marriage. The sporadic MS group included 23 age- and sex-matched cases who reported no relatives with MS or other autoimmune or neurological diseases. The first control group comprised 63 individuals in 19 families with a heterogeneous disease group: juvenile idiopathic arthritis, chronic recurrent multifocal osteomyelitis, Takayasu's arteritis, pleuroparenchymal fibroelastosis, cutis laxa, and cleft lip. The second control group included 1278 independent, ancestry-matched healthy individuals whose genomic data were provided by Dr. Elaine F. Remmers at the National Institute of Arthritis and Musculoskeletal and Skin Diseases, National Institutes of Health, from their genome-wide association study for Behçet's disease^33^. PCA of the sample is consistent with all the study participants having Turkish ancestry (Fig. 2). DNA was isolated from peripheral blood samples from the MS families, sporadic MS cases, and the first control group using the DNA Isolation Kit for Mammalian Blood (Roche) following the manufacturer’s protocol. The Ethics Committee of Istanbul University-Cerrahpasa, Cerrahpasa Faculty of Medicine approved the study (No. 83045809–604.01.02), and each individual in the study gave written, informed consent prior to sample collection. All work in this study was conducted in accordance with the Declaration of Helsinki.

### Linkage analysis, homozygosity mapping, and exome sequencing

SNP genotyping (710 K or 2.5 M, Illumina) for the MS families (24 affected and 17 unaffected; 710 K for FMS01 and FMS02 and 2.5 M for the remaining five families) and control families (63 individuals in 19 families, 710 K for all families) was performed by the Yale Center for Genome Analysis (YCGA, Connecticut, USA). Identity by descent probabilities (PI-HAT) was estimated between all pairs of individuals using the “–genome” function in PLINK 1.9^34^ to confirm family relationships and consanguineous marriages reported by the study participants (Supplementary Figures S3 and S4). Non-parametric linkage analysis was performed for each family using MERLIN^35^, and regions with LOD scores higher than 1.2 were considered candidate linkage regions. Homozygosity mapping was performed using the homozygosity detector tool of GenomeStudio (Illumina) and runs of homozygosity tool of PLINK, and regions of homozygosity > 200 kb shared exclusively by the affected family members in each family were identified. The disease status of unaffected family members younger than 50 years of age was considered “unknown” in linkage analyses, and these individuals were excluded from homozygosity mapping. Exome sequencing was performed for MS cases (N = 25) and unaffected family members older than 50 years of age (N = 13) by the Uniformed Services University, Laboratory Core of the Collaborative Health Initiative Research Program. The data were obtained in VCF format, and variants were annotated using wANNOVAR^36^. Exome variants were filtered to retain only nonsynonymous variants in coding exons and splice sites rarer than 5% frequency in gnomAD (all populations-exome) in the affected family members in each family^37^. Turkish population-specific frequencies of the variants were checked based on the work by Kars et al.^15^.

### Imputation, sum score and polygenic risk score (PRS) calculations

SNP genotypes of MS families, sporadic MS cases, control families, and population controls were used to impute ungenotyped positions across the genome using the TOPMed reference panel and imputation server^38,39^. Standard imputation quality control (QC) protocols were applied to all three datasets. Samples with a call rate < 95% and SNPs with minor allele frequency (MAF) < 5%, call rate < 95%, and p < 5 × 10^–8^ for deviation from Hardy–Weinberg expectation were excluded. Due to the small sample size, < 95% call rate was used to ensure that the maximum number of individuals were included in the study. All 24 affected and 17 unaffected family members in MS families, 23 sporadic MS cases, 63 individuals in control families, and 1272 population controls passed the pre-imputation QC step. The post-imputation QC protocol included the removal of imputed genotypes with MAF < 1% and imputation accuracy score (r^2^) of < 0.3^40^. For the weighted sum score calculation, index SNPs from the IMSGC meta-analysis study^8^ were used unless filtered for r^2^ < 0.3; in such cases, an unfiltered SNP with high linkage disequilibrium (r^2^ ≥ 0.8) with the index SNP was used. A total of 174 MS-associated SNPs (165 non-MHC, 9 MHC, Supplementary Table S6) among the 233 MS susceptibility variants and their ORs from the IMSGC were included in the sum score calculation in R using the following formula to calculate the weighted sum score for each individual:

$$Weighted\,sum\,score = \mathop \sum \limits_{i = 1}^{174} SNP_{i} \times OR_{i}$$

where SNP_i_ is coded as 0, 1, or 2 copies of the risk allele and OR_i_ is the logarithm (base 10) of the OR. Frequencies of the 174 MS-associated SNPs in the Turkish population correlate well with frequencies in gnomAD all populations and non-Finnish European populations (Pearson correlation; r = 0.932, R^2^ = 0.868, P < 0.0001; r = 0.955, R^2^ = 0.912, P < 0.0001, respectively; Supplementary Figure S5 and Supplementary Table S6). For the PRS construction, we used the discovery GWAS of MS (N = 41,505) from the IMSGC study^9^. GWAS SNPs for PRS were filtered by excluding variants with MAF < 1% and imputation quality score < 0.9, with all strand ambiguous variants and indels removed. We then constructed PRS for all subjects using a Bayesian regression framework by placing a continuous shrinkage prior on SNP effects using the PRS-CS method^41^:

$${\text{y}}N \times 1 = XN \times M\beta \times 1 + \varepsilon N \times 1$$

where y is the vector of traits, *N* denotes sample size, *M* denotes number of genetic markers, X is the genotype matrix, *β* is a vector of effect sizes of genetic markers based on OR from the GWAS, and *ε* is a vector of residual errors. PRS-CS limits the SNPs for PRS construction to approximately 1.2 million high-quality variants from the HapMap3 that provides ~ 500 SNPs per LD block, which substantially reduces computational costs. The constructed PRS values were Z-score-normalized in R to generate comparable odds ratios for subsequent downstream analyses (Supplementary Table S4)^42^. Due to the small sample size, mixed-logistic regression models using GMMAT did not converge. Therefore, logistic regression models were performed in R to compare the weighted sum score and PRS among the study groups under the hypothesis that cases would have a higher sum score and PRS compared to control families and population controls. The final results were adjusted for multiple-testing comparison using the Bonferroni correction method in R.

## Supplementary Information

Supplementary Information.

## Data Availability

The datasets and scripts generated during the current study are available from the corresponding author on reasonable request. The variants described in the study are available in the ClinVar repository with the accession IDs of SCV002072581 and SCV002072582. IMSGC datasets were provided by the Data Access Committee of IMSGC upon request.
